# Hijacking the Electron Train: Menaquinone‐Binding Antimicrobial Peptides

**DOI:** 10.1002/cbic.202500478

**Published:** 2025-08-26

**Authors:** Eilidh J. Matheson, Stephen A. Cochrane

**Affiliations:** ^1^ School of Chemistry and Chemical Engineering Queen's University Belfast David Keir Building, Stranmillis Road Belfast BT9 5AG UK

**Keywords:** antibiotics, antimicrobial peptides, menaquinone, non‐ribosomal peptides, polyprenyls

## Abstract

The rise of antibiotic resistance threatens to undermine modern medicine, making the development of new antibiotics and novel targets an urgent priority. Among emerging strategies, targeting menaquinone (MK), a membrane‐bound electron carrier, has gained traction as a promising yet underexplored approach. MK is only used for electron transport in bacteria, making it an attractive, selective antimicrobial target. Since the discovery of the first MK‐binding antimicrobial peptide (MBAMP), lysocin E, several other examples, natural and synthetic, have been reported. Given the dire need for new antibiotics that are structurally and mechanistically distinct from anything that has come before, MBAMPs potentially offer new hope in the fight against antimicrobial resistance. This review covers recent advances in the discovery, characterization, synthesis and derivatization, and mechanistic understanding of MBAMPs.

## Graphical abstract

Menaquinone is an emerging target for antimicrobial peptides. Could it be the new Achilles’ Heel of bacteria?

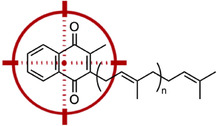

## 1 Introduction

**Figure 1:**
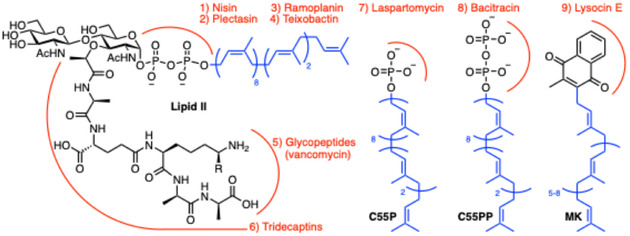
Structures of polyprenyl‐containing biomolecules (PBs) targeted by antimicrobial peptides. Undecaprenyl‐phosphate (C55P) and ‐pyrophosphate (C55PP) are utilized in multiple different bacterial glycopolymer synthesis pathways, lipid II is an intermediate in cell wall synthesis, and menaquinone (MK) is a polyprenyl quinone found only in bacteria. Red arcs denote antibiotic binding sites.

Throughout human history, infectious diseases have ravaged the human population. The black death killed half of Europe, and the Spanish Flu pandemic caused ≈50 M deaths with most dying from secondary bacterial infections.^[^ ^1^ ^]^ Antibiotics are now the cornerstone of modern medicine. The need for new antibiotic classes and targets has never been more urgent. In 2019, antibiotic‐resistant bacterial infections were responsible for an estimated 1.27 million deaths.^[^ ^2^ ^]^ The spread of resistant bacteria poses an existential threat to modern medicine and human health. Antibiotics with novel mechanisms of action offer the advantage of avoiding cross‐resistance with existing treatments. Most new antibiotics are not structurally or mechanistically distinct from existing classes (as of March 2021, less than 25% in clinical trials represent a novel class) and multidrug‐resistant bacteria continue to emerge at an alarming rate.^[^ ^3^ ^]^ Throughout the antibiotic era, membrane‐embedded biomolecules have been identified as excellent antibiotic targets for several reasons: 1) They are present on the outer leaflet of the cytoplasmic membrane, so antibiotics don’t need to permeate this membrane to access their target; 2) binding to a non‐cytosolic target prevents their ejection by efflux pumps; 3) membrane‐targeting agents do not require bacteria to be actively respiring, so can kill dormant and slow‐growing bacteria; and 4) resistance development is much lower than for other cellular processes because it is difficult for bacteria to reorganize their membranes.^[^ ^4^ ^]^ In particular, polyprenyl‐containing biomolecules feature prominently as antibiotic targets (**Figure** **1**).^[^ ^5^ ^]^ Vancomycin and ramoplanin target peptidoglycan precursor lipid II, with vancomycin binding to D‐Ala‐D‐Ala,^[^ ^6^ ^]^ and ramoplanin to the pyrophosphate.^[^ ^7^ ^,^ ^8^ ^]^ Lantibiotics^[^ 9, 10, 11, 12, 13 ^]^ (e.g., nisin^[^ ^14^ ^,^ ^15^ ^]^), defensins^[^ ^16^ ^,^ ^17^ ^]^ (e.g., plectasin^[^ ^18^ ^]^), teixobactin,^[^ ^19^ ^,^ ^20^ ^]^ and the tridecaptins^[^ ^21^ ^,^ ^22^ ^]^ also bind lipid II. Some antimicrobial peptides bind to non‐glycolipid PBs, including bacitracin^[^ ^23^ ^,^ ^24^ ^]^ (targets C55PP) and laspartomycin^[^ ^25^ ^,^ ^26^ ^]^ (targets C55P), but of particular interest is a target that was only identified in 2015, menaquinone (MK).

**Figure 2:**
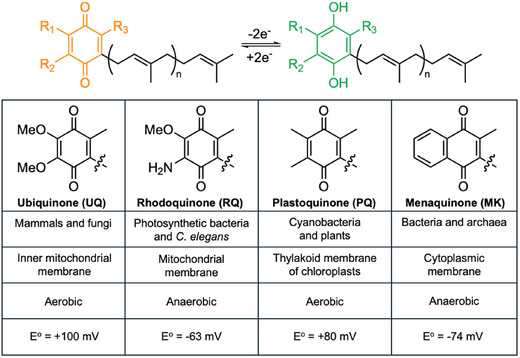
Structure and properties of different PPQs. Redox process going from quinone (orange) to quinol (green) is shown above table. Structures of quinone headgroups shown within table, along with organism in which they’re found, location in cells, oxidative process, and redox potential. Redox potentials (*E*°) obtained from refs. [28,29] Note, some bacteria may use MK under aerobic conditions for electron transport if it is the only PPQ they have.

Polyprenyl quinones (PPQs) are essential co‐factors in the electron transport chain and found across all orders of life (**Figure** **2**). The quinone ring gives PPQs their redox potential and the polyprenyl chain anchors them to the cell membrane. In Gram‐positive bacteria, MK is the only PPQ utilized,^[^ ^27^ ^]^ while facultatively anaerobic Gram‐negative bacteria also depend on MK. Ubiquinone (UQ) is found in mammals and fungi, rhodoquinone (RQ) in photosynthetic bacteria and *Candida elegans*, and plastoquinone (PQ) found in plants and cyanobacteria.^[^ ^28^ ^]^ Phylloquinone is also found in plants and has the same headgroup as MK, but contains a phytyl tail instead of a polyprenyl tail. Whereas the polyprenyl tails in PPQs vary mostly by length, the headgroups differ considerably and are responsible for their different redox potentials. Anaerobic conditions need negative redox potentials, which MK and RQ are ideal for; whereas aerobic conditions need a much greater redox potential, where UQ dominates.^[^ ^28^ ^,^ ^29^ ^]^ Although MK is found in humans, it does not play a role in cellular respiration. MK thus presents an attractive target for novel antibiotic development.

### 1.1 Lysocin E, the First of Many?

**Figure 3:**
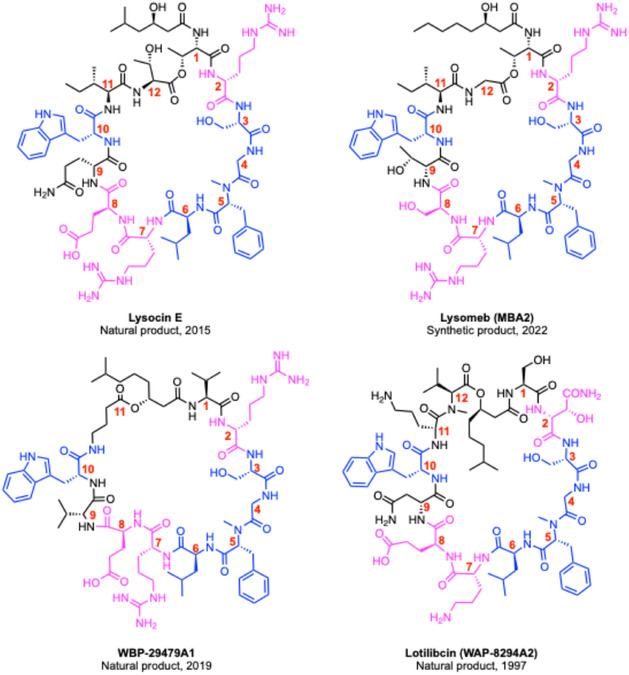
Representative structures of the four known classes of menaquinone‐binding peptides, accurate as of 15th April 2025. Conserved residues are shown in blue, and frequently conserved residues shown in magenta.

In 2015, Inoue and co‐workers reported a new streamlined approach for antibiotic discovery, unearthing the lysocin class of MBAMPs of which lysocin E (LysE) is a representative example.^[^ ^30^ ^]^ They utilized a silkworm model, allowing in vivo testing of putative antibiotics to be performed at the beginning of the discovery process.^[^ ^31^ ^,^ ^32^ ^]^ Of the 14,651 Japanese soil bacterial culture supernatants screened, 19% exhibited in vitro activity against methicillin‐sensitive *Staphylococcus aureus* (MSSA1).^[^ ^30^ ^]^ The active extracts were then injected into *S. aureus*‐infected silkworms to test their in vivo effects, and 23 cultures showed activity. Purification of one of these extracts, followed by structural determination, identified a novel cyclic lipopeptide. As it was isolated from the supernatant of *Lysobacter sp.* (RH2180−5 strain), it was aptly named lysocin E (**Figure** **3**). It is a 12‐mer depsipeptide, cyclised through the C‐terminus and the l‐Thr1 sidechain. It contains five d‐amino acids (2 x d‐Arg, d‐N‐Me‐Phe, d‐Gln, d‐Trp) and a (3*R*)‐3‐hydroxy‐5‐methylhexanoyl lipid tail. Initial testing showed that LysE had good activity (1–4 μg mL^–1^) against a range of Gram‐positive bacteria, including methicillin‐resistant *S. aureus* (MRSA). Conversely there was no activity observed against any of the Gram‐negative or fungal strains tested (*Escherichia coli* W3110, *Pseudomonas aeruginosa* PAO1, *Salmonella enterica* ATCC 14,028, *Candida albicans* ATCC 10,231, *C. tropicalis* pK233,* and *Cryptococcus neoformans* H99).

The structure of LysE did not resemble any known antibiotics, raising the question of whether it may operate by a novel mechanism of action. Hemolytic assays revealed low levels of hemolysis (<1% at 100 μg  mL^–1^), and bactericidal kinetics and membrane leakage/depolarization assays suggested the cell membrane was being targeted. Taken together, this pointed to LysE interacting with a target exclusive to bacterial membranes. There is a well‐established correlation between the presence of antibiotic resistance and temperature sensitivity in bacteria.^[^ 33, 34, 35 ^]^ Mutagenesis studies with ethyl methanesulfonate were performed on *S. aureus*, yielding temperature sensitive‐mutants that were also less susceptible to LysE. Sequencing revealed a G98A point mutation in the *menA* gene encoding a G33D amino acid substitution. MenA is a key enzyme involved in MK biosynthesis.^[^ ^36^ ^]^ It was shown that the MenA^G33D^ *S. aureus* mutant produced 40% less MK than the wild‐type strain. Phage transduction experiments confirmed that *S. aureus* isolates that gained the G98A point mutation acquired LysE resistance. *S. aureus* mutants with fully deleted *menA* or *menB* genes (also essential to MK synthesis) showed strong resistance to LysE (minimum inhibitory concentration (MIC) = 64 μg mL^–1^).^[^ ^37^ ^,^ ^38^ ^]^ 11 *S. aureus* isolates that were cultured specifically to acquire natural LysE resistance and all showed depleted MK production (<0.1–2.8% of the WT). To confirm this, antagonization assays, wherein LysE was premixed with increasing concentrations of MK, were performed and showed a dose‐dependent reduction in activity, likely due to complex formation which sequesters activity. Isothermal calorimetry (in DMSO) was then performed and showed a dissociation constant between LysE and MK of 4.1  μM. Taken together, this strongly suggests that MK is involved in the bactericidal mechanism of action of LysE. When the authors tested LysE for binding to UQ, none was observed. As the only difference between these PPQs is the substituents on carbons 5 and 6 of the quinone ring (UQ has two ‐OMe groups whereas MK has a benzene ring), this region is likely key to their selectivity. To the best of our knowledge, no MBAMPs have been tested for binding to RQ or PQ.

Further work focused on *Mycobacterium tuberculosis*, which caused 1.3 million deaths in 2022. MK is the exclusive enzyme co‐factor in mycobacterial electron transport chains, so mutations that make it less susceptible to LysE would also be deleterious to its growth. LysE showed potent activity against a range of *M. tuberculosis* strains, including multiple multi‐drug‐resistant isolates (MIC = 0.13–0.5 μg mL^–1^).^[^ ^39^ ^]^ Like with *S. aureus*, premixing MK with LysE reduced activity. Monitoring the kill kinetics over 21 days showed LysE had killed the entire visible population of *M. tuberculosis* by day 15, and crucially, that the level of *M. tuberculosis* did not rise again in the remaining 6 days. None of the clinically prescribed isoniazid, rifampicin, or bedaquilline reduced the *M. tuberculosis* to below detectable levels and after reaching a minimal level, the population of bacteria increased again. A major hurdle to overcome in the treatment of tuberculosis is the ability to target dormant *M. tuberculosis*.^[^ ^40^ ^]^ Many clinically available drugs can only kill actively replicating populations. When tested against dormant non‐replicating persistent *M. smegmatis* at 3 x MIC concentration, lysocin reduced the population by 3.6log_10_ colony‐forming units (CFUs). It outperformed rifampicin and bedaquilline significantly (reductions of 0.66log_10_ CFU and 0.22log_10_ CFU, respectively). This work shows the great promise offered by LysE as an anti‐tuberculosis drug. In vivo mouse model studies have been conducted with LysE for both *S. aureus* and *M. tuberculosis*.^[^ ^30^ ^,^ ^39^ ^]^ The median effective dose (ED_50_) against *S. aureus* was 0.5 mg kg^–1^, which is lower than that of vancomycin, and no toxicity was detected at dose of up to 400 mg kg^–1^. In a mouse model of *M. tuberculosis* infection, LysE was administered subcutaneously at 40 mg kg^−1^ daily, 5 days per week for 2 weeks. This regimen led to a significant reduction in CFUs in the lungs, spleen, and liver compared to untreated controls.

**Scheme 1:**
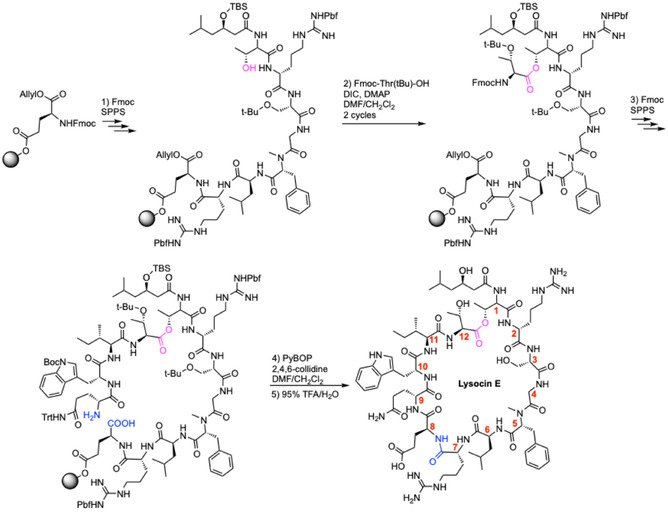
Total synthesis of lysocin E.^[^ ^41^ ^]^ DIC = Diisopropylcarbodiimide, DMAP = 4‐dimethylaminopyridine, DMF = dimethylformamide, Pbf = 2,2,4,6,7‐pentamethyldihydrobenzofuran‐5‐sulfonyl, PyBOP = (1*H*‐1, 2, 3‐benzotriazol‐1‐yloxy)‐tris(pyrrolidino)‐phosphonium hexafluoro phosphate, SPPS = solid‐phase peptide synthesis, TBS = *tert*‐butyl(dimethyl)silyl, TFA = trifluoroacetic acid, Trt = trityl.

The first total synthesis of LysE was reported in 2015 by Inoue and co‐workers (**Scheme** **1**).^[^ ^41^ ^]^ The starting point for the synthesis was Fmoc‐l‐Glu‐OAllyl, immobilized to Wang resin through the sidechain carboxylic acid. From there, peptide elongation was achieved through Fmoc SPPS (amino acid, HBTU, HOBt, DIPEA, DMF/*N*‐methyl‐2‐pyrrolidone). Esterification was achieved with Fmoc‐l‐Thr(tBu)‐OH, DIC and DMAP in CH_2_Cl_2_ and DMF. Once the linear peptide chain was completed, on‐resin cyclization was performed with PyBOP, 2,4,6‐collidine in CH_2_Cl_2_ and DMF. Resin cleavage, global deprotection, and HPLC purification gave LysE in 8% overall yield. Out of all known MBAMPs, the most significant structure–activity relationship (SAR) studies have been performed on LysE.^[^ ^41^ ^,^ ^42^ ^]^ As well as reporting the first total synthesis of LysE, Inoue and co‐workers synthesized the enantiomeric form of this peptide, *ent*‐LysE. This is a common strategy employed in peptide SAR because if the enantiomer retains full activity, this usually suggests an achiral target. *Ent*‐LysE retained activity comparable to the natural peptide, suggesting an achiral target and strengthening the case for MK‐binding being important in the peptide's mechanism of action. In this study they also synthesized an analogue missing the *N*‐methyl group at position 5, LysE(d‐Phe5), which was found to have eightfold lower activity than LysE. In contrast, an analogue in which the chirality of the N‐terminal fatty acid *β*‐hydroxyl had been inverted (*S*FA‐LysE) retained activity, showing lipid tail modifications are tolerated. This is common for many different non‐ribosomal antimicrobial peptides.^[^ 43, 44, 45, 46, 47 ^]^ Following this study, Inoue and coworkers followed up with another SAR study in which 2401 LysE analogues were evaluated.^[^ ^42^ ^]^ They used the split‐pool technique to rapidly prepare 2401 resin bound peptides in which positions 3, 6, 9, and 11 were varied. A high‐throughput screening (HTS) approach was used to select for active peptides (see reference^[^ ^42^ ^]^ for full details) and 32 novel analogues were evaluated. Position 3 modifications (l‐Ser in LysE) were well tolerated, with substitutions including l‐Ala, l‐Asn, l‐Asp, l‐Tyr, and l‐Val resulting in similar activity to the natural peptide against MSSA1. In contrast, position 6 modifications were not well tolerated, with any deviations from the natural l‐Leu (e.g., l‐Ala, l‐Ser, or l‐Tyr) killing activity. Position 9 could be changed from d‐Asn to d‐Orn, d‐Ser, d‐Tyr, or d‐Val without harming potency, but almost all position 11 from natural l‐Ile (l‐Tyr was the exception) were detrimental. It's important to note that the screening approach used meant that most analogues were not actually screened for activity (only analogues identified as MK‐binders in the HTS assay were screened), but this work still highlights that positions 3 and 9 can be modified, whilst positions 6 and 11 are crucial for activity.

### 1.2 WAP‐8294A2, Just Rolls off the Tongue

The discovery of LysE cemented MK as a valid target for antibiotics and led to a re‐examination of known antimicrobial peptides whose targets had never been determined. Of particular interest was any that were isolated from *Lysobacter* (the producer of LysE). In 1997, the cyclic depsipeptide WAP‐8294A2 (Figure 3) was isolated from *Lysobacter* spp.^[^ ^48^ ^]^ and showed strong activity against MRSA isolates (MIC = 0.78 μg mL^−1^).^[^ ^49^ ^]^ Although there are distinct structural differences between WAP‐8294A2 and LysE, such as the macrocycle size and the nature of the ester linkage, there are also many similarities. Both contain twelve amino acids and a *γ*‐hydroxy lipid tail, with six conserved residues: l‐Ser‐3, Gly‐4, *N*‐Me‐d‐Phe‐5, l‐Leu‐6, l‐Glu‐8, and d‐Trp‐10. Like LysE, WAP‐8294A2 showed activity against Gram‐positive bacteria but was not active against Gram‐negative bacteria and fungi. Knocking out the *menA* or *menB* genes in *S. aureus* imparted resistance against WAP‐8294A2, and fluorescent leakage assays with large unilamellar vesicles (LUVs) containing MK showed that all peptides induced membrane damage in a dose dependent manner. This strongly suggested that like LysE, the WAP‐8294A2‐MK interaction is key to its bactericidal activity. Further in vivo studies showed good efficacy against MRSA infections in mouse models.^[^ ^49^ ^]^ The ED_50_ was determined to be 0.38 mg kg^–1^, in comparison the ED_50_ of 5.3 mg kg^–1^ that was determined for vancomycin. No toxicity was observed up to 200 mg kg^–1^. WAP‐8294A2 is also known as lotilbicin, and in 2004, aRigen Pharmaceuticals initiated a U.S. Phase I clinical trial to evaluate the safety and tolerability of WAP‐8294A2 in healthy volunteers. This single‐dose, double‐blind, placebo‐controlled study enrolled 40 participants. However, there is limited publicly available information regarding the progression of WAP‐8294A2 beyond this initial Phase I trial. As of now, there are no records of subsequent clinical trials or approvals for this compound and the compound's development status remains uncertain.

**Scheme 2:**
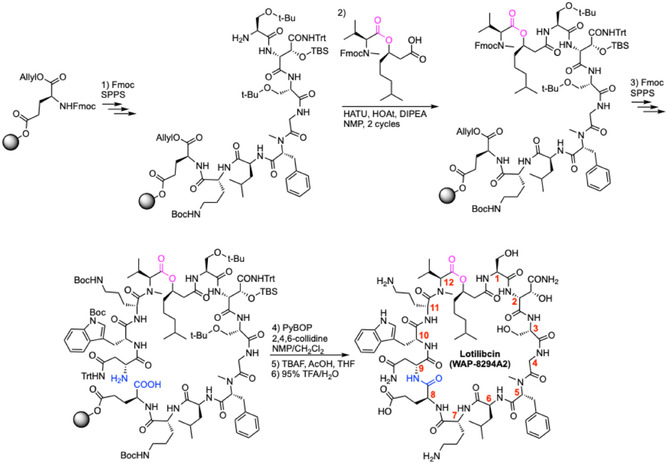
Total synthesis of WAP‐8294A2.^[^ ^50^ ^]^ HATU = 2‐(7‐Aza‐1‐*H*‐benzotriazol‐1‐yl)‐1,1,3,3‐tetramethylaminium hexafluorophosphate, HOAt = 1‐hydroxy‐7‐azabenzotriazole, NMP = *N*‐methyl‐2‐pyrrolidone, PyBOP = (1*H*‐1, 2, 3‐benzotriazol‐1‐yloxy)‐tris(pyrrolidino)‐phosphonium hexafluoro phosphate, SPPS = solid‐phase peptide synthesis, TBS = *tert*‐butyl(dimethyl)silyl, TFA = trifluoroacetic acid, and Trt = trityl.

To determine if WAP‐8294A2 and LysE shared a common target, Inoue and co‐workers first completed the total synthesis of WAP‐8294A2, and a deoxy analogue, WAP‐8294A2 (d‐Asn‐2) (**Scheme** **2**).^[^ ^50^ ^]^ The starting point for the synthesis was Fmoc‐l‐Glu‐OAllyl, immobilized to Wang resin through the sidechain carboxylic acid. From there, peptide elongation was achieved through Fmoc SPPS (amino acid, HATU, HOAt, and DIPEA in NMP). Rather than perform the esterification reaction on‐resin, as seen in the synthesis of LysE (Scheme 1), the necessary building block containing the *N*‐Me‐l‐Val‐12 (*R*)‐3‐hydroxy‐7‐methyl‐octanoyl ester was pre‐assembled via solution‐phase chemistry and then coupled to the immobilized peptide. Once the linear peptide chain was completed, on‐resin cyclization was performed with PyBOP and 2,4,6‐collidine in CH_2_Cl_2_/NMP. Resin cleavage, global deprotection, and HPLC purification gave WAP‐8294A2 in 8% overall yield. With WAP‐8294A2 and WAP‐8294A2 (d‐Asn‐2) in hand, their activity was determined against a panel of bacteria and fungi.^[^ ^50^ ^]^ SAR studies have also been conducted on WAP‐8294A2 by Li and coworkers.^[^ ^51^ ^]^ Consistent with SAR work on LysE, the authors found that the enantiomer of WAP‐8294A2 (*ent*‐ WAP‐8294A2) retained full activity, providing further evidence that binding to the achiral target MK is important for antimicrobial activity. Additionally, it was shown that the chirality of the hydroxyl group on d‐2OH‐Asn can be inverted, or removed entirely, without any loss in activity. An analogue missing the *N*‐methyl group at position 12, WAP‐8294A2(l‐Val12) was completely inactive, highlighting the importance of this methyl unit. An alanine scan was also conducted, showing that residues 4, 5, 6, 7, 10, and 11 are essential for WAP‐8294A2 activity. Residues 4–7 are the Gly‐d‐*N*MePhe‐l‐Leu‐d‐Asn/Orn tetrad that is highly conserved across all known MBAMPs. As well as an alanine scan, a lysine scan showed that modifications at positions 3, 7, 8, 9, and 11 were tolerated, but not at position 6 (l‐Leu in natural peptide). Conservative substitutions of ornithines are positions 7 and 11 with other cationic amino acids were well tolerated, but any attempt to substitute d‐Trp10 was unsuccessful.

### 1.3 WBP‐29,479A1, the Naming Trend Continues

In 2019, a new MBAMP, WBP‐29,479A1 (Figure 3), was reported by Du and co‐workers.^[^ ^52^ ^]^ Genome mining of *Lysobacter* species led to the discovery of putative non‐ribosomal peptide synthases (NRPSes) wbpA and wbpB in a *Lysobacter antibioticus* strain. Analysis of *wbpA* and *wbpB* genes using antiSMASH^[^ ^53^ ^]^ predicted that they encode NRPSes that produce an 11 amino acid peptide that was N‐terminally acylated. Extensive LC‐HRMS screening of metabolites from *L. antibioticus* cultures grown in different conditions identified the potential peptide product produced by these NRPSes (m/z 743 for [M + 2H]^2+^). Metabolite screening of *L. antibioticus* (Δ*wbp*) could not identify the suspected product, indicating that the *wbp* biosynthetic gene cluster (BCG) was responsible for the identified product. Isolation and characterization revealed this product to be a cyclic depsipeptide composed of 11 amino acids, cyclized through the C‐terminus and the *γ*‐hydroxyl group of the lipid tail. This peptide contained the six residues observed in both LysE and WAP‐8294A2: l‐Ser‐3, Gly‐4, *N*‐Me‐d‐Phe‐5, l‐Leu‐6, l‐Glu‐8, and d‐Trp‐10. It carried a similar activity profile to that of lysocin and WAP‐8294A2, strong activity against a range of Gram‐positive bacteria but poor activity against Gram‐negative bacteria or fungi. Antagonization assays with *S. aureus* showed that peptide activity dramatically decreased in the presence of MK but not UQ. To date, no total synthesis of WBP‐29479A1 has been reported.

### 1.4 Bioinspired MBAMPs

**Scheme 3:**
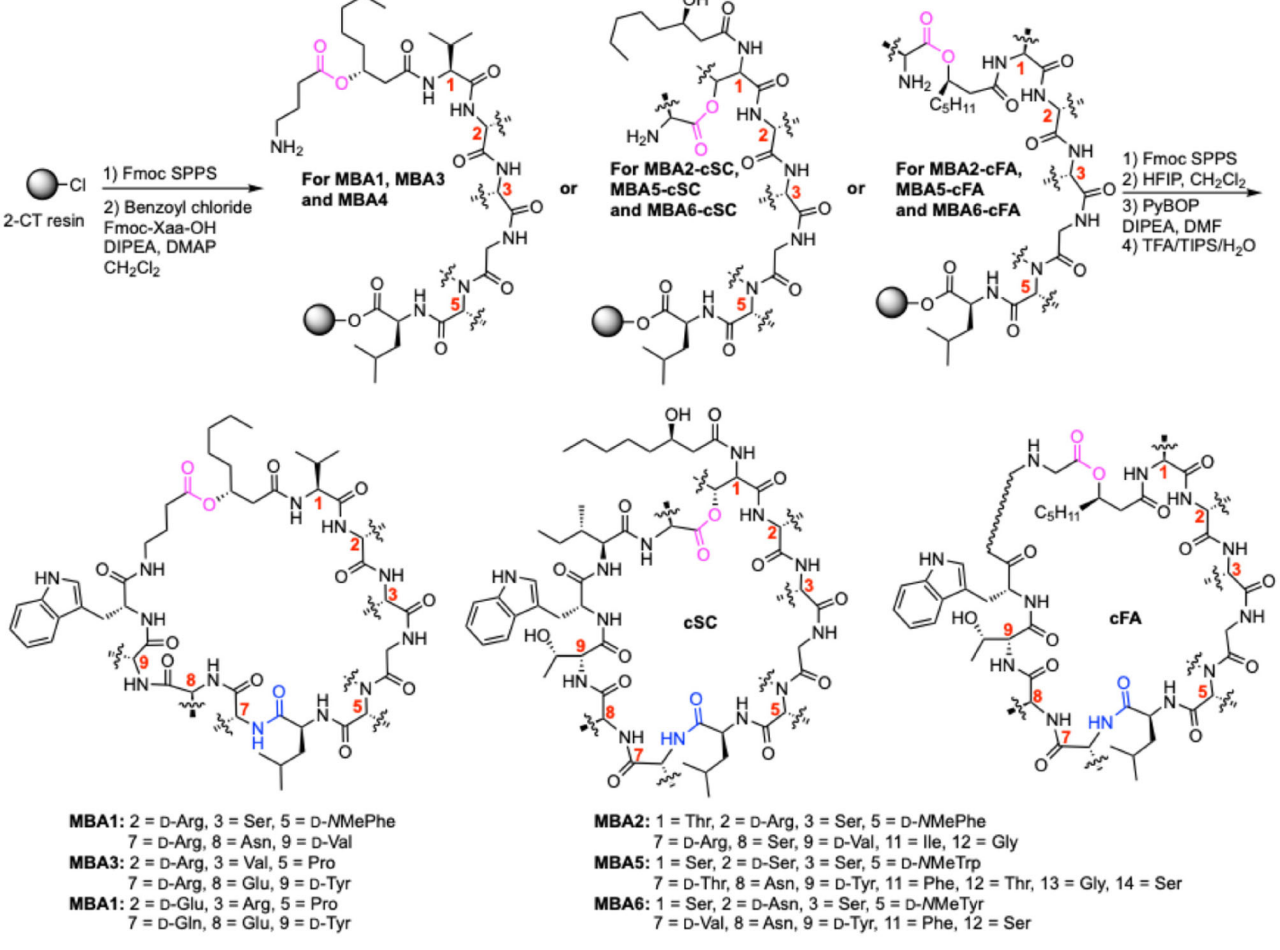
Total synthesis of MBA analogues.^[^ ^54^ ^]^ cFA = cyclized on fatty acid, cSC = cyclized on side chain, DIPEA = diisopropylethylamine, DMF = dimethylformamide, HFIP = hexafluoroisopropanol, PyBOP = (1*H*‐1, 2, 3‐benzotriazol‐1‐yloxy)‐tris(pyrrolidino)‐phosphonium hexafluoro phosphate, SPPS = solid‐phase peptide synthesis, Xaa = amino acid, TFA = trifluoroacetic acid, and TIPS = triisopropylsilane.

In 2022, Brady and co‐workers reported a series of “menaquinone‐binding antibiotics” (MBAs).^[^ ^54^ ^]^ They screened soil metagenomic libraries for genes encoding adenylation domains (A domains) corresponding to the six conserved residues (l‐Ser‐3, Gly‐4, *N*‐Me‐d‐Phe‐5, l‐Leu‐6, l‐Glu‐8, and d‐Trp‐10) found in LysE, WAP‐8294A2, and WBP‐29479A1. When this approach failed to identify any novel peptides, they widened the search for the motif Gly‐X‐Leu‐X–X–X–Trp, returning six potential BCGs. Given the challenges and complexity of secondary metabolite production and extraction, the decision was made to pursue structure prediction and total chemical synthesis as the primary approach for generating new MBAMPs. This approach has been well documented by the Brady group for other non‐ribosomal peptides to produce what they call “synthetic‐bioinformatic natural products (syn‐BNPs).”^[^ 55, 56, 57 ^]^ The specificity of adenylation (A) domains and the absence of tailoring enzymes in most BCGs enabled confident structural predictions to be performed. However, these BGCs did not reveal details about cyclization or lipid tail identity. Given the structural tolerance observed in other MBAMPs, and the presence of (*R*)‐3‐hydroxy‐octanoic acid in WAP‐8294A, this lipid tail was used in the synthesis of all predicted peptides. Cyclization was achieved via the *β*‐hydroxyl group on the fatty acid (cFA) or nucleophilic side chain of the N‐terminal amino acid (cSC) (**Scheme** **3**).^[^ ^54^ ^]^ Fmoc‐SPPS was used to prepare the linear peptide chain, starting with resin‐bound l‐Leu‐6. The ester bond was installed using a Yamaguchi esterification (amino acid, benzoyl chloride, DIPEA, and DMAP in CH_2_Cl_2_ for 3 days). Following full chain elongation, protected peptides were cleaved from resin and cyclized using PyAOP and DIPEA in DMF. This process yielded ten potential MBAMPs, which were then tested for activity against *Bacillus subtilis*, *S. aureus*, and *S. epidermidis*. In most cases, cyclization through the N‐terminal residue's side chain resulted in higher potency than cyclization through the N‐terminal fatty acid. This ultimately led to the identification of six novel antimicrobial peptides, MBA1–MBA6. Notably, the MBA4, MBA5, and MBA6 sequences originated from *Paracoccus alcaliphilus*, *Dyella tabacisoli*, and *Dyella mobilis*, respectively, marking the first instance of non‐Lysobacter strains producing MBAMPs.^[^ ^54^ ^]^

MBA1–MBA6 were evaluated against various Enterococcus and Streptococcus strains.^[^ ^54^ ^]^ While active against MK‐producing strains, they showed no activity against strains deficient in MK, indicating a specific MK‐targeting mechanism. Time‐kill assays with *S. aureus*, membrane depolarization studies, and fluorescence leakage assays confirmed bactericidal activity via membrane lysis. All six peptides lost activity against *S. aureus* ΔmenA and ΔmenB mutants. Supplementing MIC media with MK, but not UQ, increased MICs in a dose‐dependent manner. Resistance studies revealed point mutations in MK biosynthesis genes in resistant *S. aureus strains*. Isothermal titration calorimetry (ITC) with liposomes containing MK showed dissociation constants between 0.09 and 0.30 μM for peptides, with no binding observed to UQ.^[^ ^54^ ^]^ These findings underscore the MBAMPs specific and potent interaction with MK, leading to bacterial cell death. Peptides exhibited activity exclusively against Gram‐positive bacteria. Although facultatively anaerobic Gram‐negative bacteria utilize MK, their MK levels peak under anaerobic conditions. Testing the six peptides against *E. coli* BAS849 (a strain with a permeable outer membrane) under anaerobic conditions yielded MICs ranging from 8 to 32 μg mL^−1^. Under aerobic conditions, MICs increased two‐ to four‐fold. This represents the first demonstration of MBAMP activity against a Gram‐negative strain, albeit under specific conditions. The outer membrane barrier likely contributes to MBAMP inactivity against Gram‐negative bacteria. When tested against six *Mycobacterium tuberculosis* strains, all MBAs except MBA5 and MBA6 showed moderate to strong activity. MBA3 notably achieved an MIC of 0.078 μg mL^−1^ against multidrug‐resistant *M. tuberculosis*. Given the ability of *M. tuberculosis* to replicate within macrophages, effective agents must target intracellular bacteria. MBA1, MBA2, and MBA3 demonstrated the ability to kill *M. tuberculosis* within mouse macrophages, with IC_5__0_ values ranging from 0.14 to 2.1 μg mL^−1^, highlighting their potential as anti‐tuberculosis agents. In MRSA mouse models, subcutaneous administration of 10 mg kg^−1^ MBA3 and 30 mg kg^−1^ MBA6 resulted in 100% survival rates.^[^ ^54^ ^]^ Building on this approach, Li and colleagues screened 14,298 bacterial genomes for genes encoding A‐domains encoding the Gly‐X‐Leu‐X‐X‐X‐Trp motif, as well as shorter motifs like Gly‐X‐Leu and Leu‐X‐X‐X‐Trp.^[^ ^58^ ^]^ They identified two BGCs potentially representing a new MBAMP structural class: MBA7 from *Dyella sylvatica* and MBA8 from *Pseudomonas* sp. These peptides, synthesized as previously described, were renamed silvmeb and pseudomeb, respectively. Both exhibited MICs of 4–8 μg mL^−1^ against various Gram‐positive bacteria but lacked activity against Gram‐negative bacteria and fungi. MK‐targeting by silvmeb and pseudomeb was confirmed through *S. aureus* time‐kill assays and membrane depolarization studies. Adding MK to MIC media reduced their potency in a dose‐dependent manner. Resistance studies yielded three *S. aureus* strains with mutations in MK biosynthesis genes. In MRSA mouse models, both peptides achieved 100% survival rates at doses of 25 mg kg^−1^.

## 2 Mechanism of Action

Recent studies on the AMPs teixobactin^[^ ^59^ ^]^ and plectasin^[^ ^18^ ^]^ have shown that the mechanism of action of non‐ribosomal peptides that bind to non‐protein membrane targets are often highly complex. Rarely is it as simple as 1:1 antibiotic:target binding kills bacteria. It can take years of intricate experiments to unravel these complicated mechanisms, which will likely also be the case for MBAMPs. The studies described above on LysE, WAP‐8294A2, WBP‐29479A1, and synthetic MBAMPs conclusively show that these peptides selectively bind to MK with nM binding affinities (determined by ITC), but not UQ. This binding then results in membrane disruption, which ultimately kills bacteria. However, how peptides bind to MK, whether any additional membrane lipids (e.g., lipid II) are important, or how MK‐binding leads to membrane disruption and cell death, are unclear. Regarding the binding mechanism of peptides to MK, some form of *π*–stacking interaction may be involved between the d‐Trp residue conserved across all peptides, and the MK head group, but without an X‐ray diffraction or NMR‐derived structure, this is only speculation. Given the prevalence of lipid II binding antimicrobial peptides,^[^ ^5^ ^]^ investigations were performed to assess MBAMP‐lipid II binding. Immobilisation studies by Walker and co‐workers on LysE suggested a stronger binding affinity for lipid II than MK.^[^ ^60^ ^]^ However, Hamamoto and co‐workers observed that liposomes containing lipid II do not undergo significant membrane disruption upon the addition of LysE, and liposomes that contain MK only show comparable membrane disruption to those containing both MK and lipid II upon LysE exposure.^[^ ^61^ ^]^ In mouse infection models, MBAMPs have a lower therapeutic dose than vancomycin, despite having a higher MIC.^[^ ^30^ ^]^ This intriguing phenomenon indicated that there could be a host factor in the mouse that is amplifying the MBAMP potency. This theory is supported by the observation that the addition of bovine serum increases the activity of LysE in vitro.^[^ ^42^ ^]^ Modification of activity by host factors is observed throughout antimicrobials: sometimes beneficial, sometimes deleterious. Through activity‐guided fractionation, Hamamoto and co‐workers determined the known apoproteins bovine ApoA‐I and bovine ApoA‐II as the host factors in bovine serum increasing the activity of LysE.^[^ ^61^ ^]^ The same activity increase was observed when either human or mouse ApoA‐I and ApoA‐II were used. Taken together, Hamamoto and co‐workers proposed that LysE initially binds to MK, inflicting damage to the membrane, before then binding to lipid II, which enhances affinity for Apo‐I/II, and finally binding to Apo‐I/II, inflicting catastrophic membrane activity and enhancing the bactericidal effect. SAR work has shown that the enantiomers of LysE^[^ ^41^ ^]^ and WAP‐8294A2^[^ ^51^ ^]^ retain full activity, so direct MBAMP‐lipid II binding is not occurring, otherwise the enantiomers would show much lower activity as lipid II is chiral. When Brady and co‐workers observed binding interactions through ITC of MBAMPs and lipid II, they then tested inactive MBAMP analogues and found they also showed lipid II binding.^[^ ^54^ ^]^ Lipid II binding may therefore be a non‐specific interaction that is not crucial to the bactericidal activity of MBAMPs but more work is required to unpick this complex interaction.

In 2025, our own group developed a semi‐synthetic method to attach chemical labels (fluorophores, affinity tags, etc.) at the *ω*‐terminus of the PPQs UQ and MK.^[^ ^62^ ^]^ This labeling strategy afforded PPQs that retained their natural headgroup and polyprenyl chain length, with the label placed in an innocuous position far from the enzyme/peptide binding site. Fluorescently labeled UQ and MK were used to study the effect of MBAMPs on model membranes. Giant Unilamellar Vesicles (GUVs) composed of 98% dioleoylphosphatidylcholine (DOPC), 2% unlabeled PPQ, and 0.1% BODIPY‐PPQ were prepared and treated with an analogue of lysocin E, or MBA2. For GUVs containing just UQ, no effect was observed on membranes, even after 1 h. However, addition of MBAMP to MK‐containing vesicles resulted in rapid MK aggregation and vesiculation, eventually resulting in total membrane rupture within 1 h. ITC measurements were also performed, with LUVs composed of 1:1 dioleoylphosphatidylglycerol (DOPG) (DOPC) +1.25% MK titrated into MBAMP. MBAMPs bound strongly to these MK‐containing LUVs, with binding affinities (*k*_D_) in the range of 59–80 nm. This suggests that the binding interaction between MBAMPs and MK only involves the menadione headgroup, as MBAMPs retained nM binding affinities to MK analogues containing bulky chemical labels (e.g., BODIPY or NBD) at the end of their polyprenyl tail. ITC studies also revealed that rapid peptide aggregation occurs in the presence of just a small amount of MK, consistent with GUV studies. This localization of non‐protein lipid targets upon AMP binding is also observed when the AMPs plectasin, copsin, nisin, teixobactin, ramoplanin, and teicoplanin bind to lipid II.^[^ ^18^ ^]^ At a macromolecular level, MBAMPs clearly cause MK aggregation and membrane rupture. The exact binding mechanism of MBAMP to MK that initiates this destructive cascade remains to be elucidated.

### 2.1 Peptide Promises and Pitfalls

There is a desperate need for new antibiotics that are structurally and mechanistically distinct from anything that has come before. One could argue that in the age of antimicrobial resistance, non‐protein membrane targets are preferrable, given that DNA mutations do not directly result in target modification, and their presence on the membrane surface precludes the need for membrane permeabilization (a common hurdle in drug discovery). MK offers a possibility beyond the usual peptidoglycan intermediate class of targets (C_55_P, C_55_PP, lipid I, and lipid II),^[^ ^5^ ^]^ which although less prone to resistance development, are certainly not resistance proof. The efficacy of MBAMPs has been shown in vivo (murine infection models) against S. aureus and M. tuberculosis.^[^ ^30^ ^,^ ^39^ ^]^ Although current studies show low cytotoxicity, to date details on nephrotoxicity have not been reported. This is an important consideration for peptide antibiotics, as the main drawbacks of both clinically used peptide antibiotics, daptomycin and polymyxin, are their nephrotoxicity. The low cytotoxicity observed with MBAMPs, despite mammalian extrahepatic cells also containing MK, is likely due to their inability to reach MK on mitochondrial membranes due to limited access across mammalian plasma membranes. MBAMPs are effective against *M. tuberculosis,* one of the four World Health Organization's “critical priority pathogens”. The other critical priority pathogens (carbapenem‐resistant Enterobacterales, third generation cephalosporin‐resistant Enterobacterales and *Acinetobacter baumannii*) are intrinsically resistant to current MBAMPs, which cannot cross the outer membrane of Gram‐negative bacteria. Therefore, although the MBAMPs unique mechanism of action makes them a promising class of new antibiotics, a more rigorous evaluation of their toxicity, and expansion of their activity to Gram‐negative pathogens, likely needs to be realized before they can be considered beyond pre‐clinical trials.

## Conflict of Interest

The authors declare no conflict of interest.
